# Advancements and limitations of image‐enhanced endoscopy in colorectal lesion diagnosis and treatment selection: A narrative review

**DOI:** 10.1002/deo2.70141

**Published:** 2025-05-08

**Authors:** Taku Sakamoto, Shintaro Akiyama, Toshiaki Narasaka, Kiichiro Tuchiya

**Affiliations:** ^1^ Division of Gastroenterology University of Tsukuba Hospital Ibaraki Japan

**Keywords:** artificial intelligence, colorectal cancer, image‐enhanced endoscopy, invasion depth assessment, ulcerative colitis‐associated neoplasia

## Abstract

Colorectal cancer (CRC) is a leading cause of cancer‐related mortality, highlighting the need for early detection and accurate lesion characterization. Traditional white‐light imaging has limitations in detecting lesions, particularly those with flat morphology or minimal color contrast with the surrounding mucosa. It also struggles to distinguish neoplastic from non‐neoplastic lesions. These limitations led to the development of image‐enhanced endoscopy (IEE). Image‐enhanced endoscopy modalities such as narrow‐band imaging, blue laser imaging, linked color imaging, and texture and color enhancement imaging enhance mucosal surface and vascular pattern visualization, thereby improving lesion detection and characterization.

In contrast, red dichromatic imaging is primarily designed to enhance the visibility of deep blood vessels, making it particularly useful during therapeutic endoscopies, such as identifying bleeding sources and monitoring post‐treatment hemostasis. Although IEE enhances lesion detection and characterization, it remains limited in assessing submucosal invasion depth, which is a key factor in treatment decisions. Endoscopic submucosal dissection requires accurate prediction of invasion depth; however, IEE mainly reflects superficial features. Endoscopic ultrasound and artificial intelligence‐assisted diagnostics have emerged as complementary techniques for improving depth assessment and lesion classification. Additionally, IEE plays a critical role in detecting ulcerative colitis‐associated neoplasia (UCAN), which often presents with a flat morphology and indistinct borders. High‐definition chromoendoscopy and IEE modalities enhance detection; however, inflammation‐related changes limit diagnostic accuracy. Artificial intelligence and molecular biomarkers may improve UCAN diagnosis. This review examines the role of IEE in lesion detection and treatment selection, its limitations, and complementary techniques such as endoscopic ultrasound and artificial intelligence. We also explored pit pattern diagnosis using crystal violet staining and discussed emerging strategies to refine colorectal cancer screening and management.

## INTRODUCTION

**FIGURE 1:**
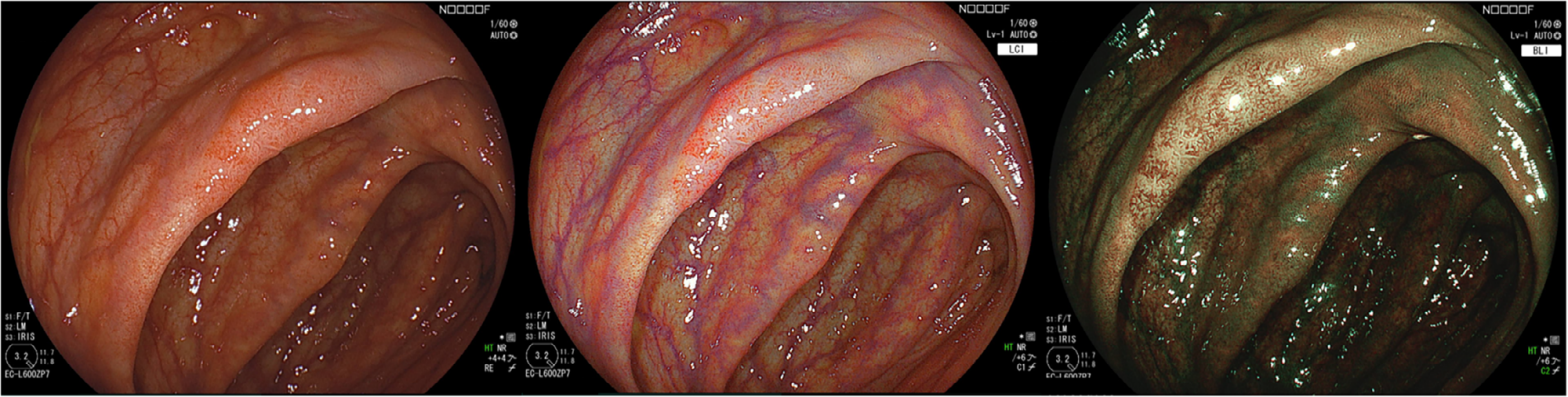
Differences in lesion visibility among white‐light imaging, linked color imaging, and blue laser imaging. Linked color imaging enhances redness in tumor areas, improving lesion recognition and providing greater overall brightness, which increases the amount of information visible in the field of view. In contrast, blue laser imaging emphasizes vascular and surface patterns, making it particularly useful for identifying lesion characteristics.

**FIGURE 2:**
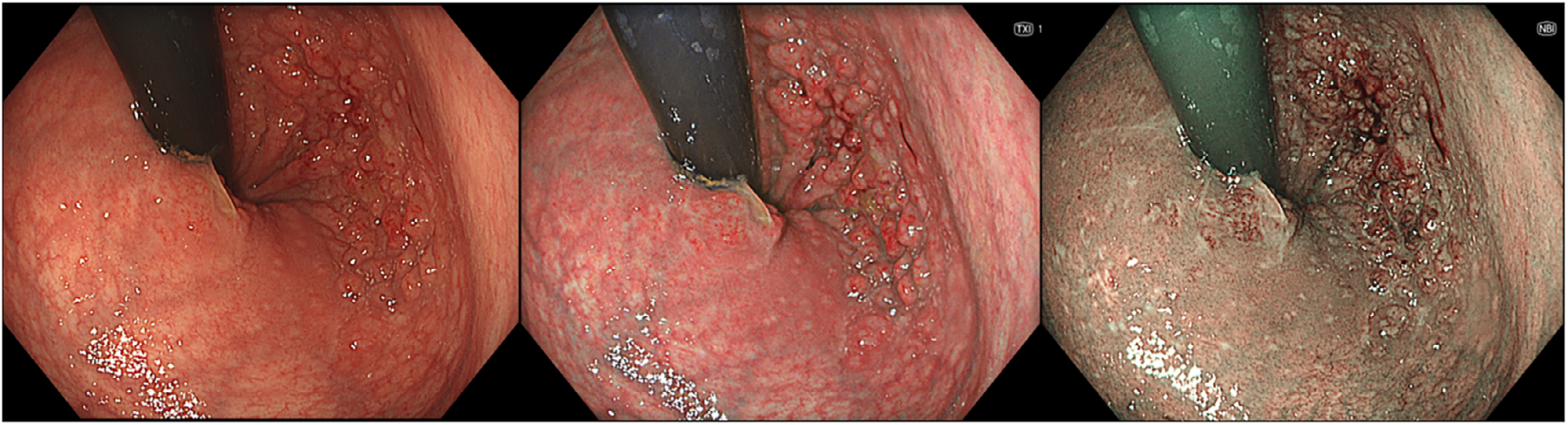
Comparison of white‐light imaging, texture and color enhancement imaging, and narrow‐band imaging, demonstrating how each modality enhanced lesion visibility

Colorectal cancer (CRC) is one of the leading causes of cancer‐related mortality worldwide, and early detection and accurate characterization of colorectal lesions are essential for improving patient outcomes.^1^, ^2^ Because of its limited ability to detect lesions^3^ especially flat lesions or those with poor color contrast against the surrounding mucosa and to differentiate neoplastic from non‐neoplastic tissue, traditional white‐light imaging (WLI) prompted the development of image‐enhanced endoscopy (IEE). IEE encompasses several advanced imaging modalities such as narrow‐band imaging (NBI), blue laser imaging (BLI), linked color imaging (LCI), and texture and color enhancement imaging (TXI) that primarily aim to improve lesion detection and characterization (Figures 1 and 2).^4^, ^5^, ^6^, ^7^, ^8^, ^9^ In addition, red dichromatic imaging (RDI) is designed to enhance the visibility of deeper blood vessels and is mainly utilized during therapeutic procedures, such as hemostasis and post‐treatment monitoring.

Although IEE has demonstrated significant improvements in lesion detection rates and the characterization of mucosal patterns, its effectiveness is inherently limited in assessing tumor invasion depth. As endoscopic submucosal dissection (ESD) has become increasingly utilized for en‐bloc resection of early‐stage colorectal tumors, the need for accurate prediction of submucosal invasion depth has become a critical factor in treatment decision‐making.^10^, ^11^, ^12^ However, IEE primarily reflects superficial mucosal features and lacks the ability to provide objective information regarding the depth of tumor infiltration, which is crucial for distinguishing between lesions suitable for endoscopic resection and those requiring surgical intervention.

To address this limitation, alternative diagnostic modalities, such as endoscopic ultrasound (EUS), have been explored for assessing submucosal invasion.^13^, ^14^ Additionally, the integration of artificial intelligence (AI)‐assisted diagnostics has shown promise in improving the objectivity and accuracy of lesion classification.^15^, ^16^ Despite these advancements, there remains a need for further validation and standardization of multimodal imaging approaches to optimize the treatment selection for colorectal lesions.

Furthermore, IEE plays a crucial role in the detection and diagnosis of ulcerative colitis‐associated neoplasia (UCAN), which is challenging because of its multifocal nature, flat morphology, and indistinct borders^17^. Conventional WLI often fails to detect UCAN, leading to the recommendation of high‐definition chromoendoscopy or IEE techniques, such as NBI and TXI, to enhance lesion visualization^18^. However, chronic inflammatory changes in ulcerative colitis (UC) can obscure neoplastic transformations, thereby limiting the diagnostic accuracy of IEE alone. The integration of AI‐based image analysis and molecular biomarkers may offer a solution for improving UCAN detection in the future.

This review examined the diagnostic accuracy of IEE for both lesion detection and treatment selection. We discussed its strengths and limitations, the role of complementary techniques, such as EUS and AI, and its application in UCAN diagnosis. Finally, we highlighted emerging strategies to overcome the current limitations of IEE in CRC screening and management.

## OVERVIEW OF IEE TECHNOLOGIES

### Key IEE modalities

This section outlines the key IEE modalities and their primary clinical applications, including not only diagnostic enhancements but also therapeutic support, as exemplified by RDI. Narrow‐band imaging enhances vascular and surface pattern recognition using specific‐wavelength light filtration, allowing improved differentiation between neoplastic and non‐neoplastic lesions. Blue laser imaging employs a laser‐based light source with a narrow bandwidth that enhances the mucosal and vascular contrast, making it particularly useful for detecting early CRC. Linked color imaging enhances mucosal color contrast, which may facilitate the identification of flat and serrated lesions that are often challenging to detect using conventional WLI. Texture and color enhancement imaging is a newer modality developed by Olympus that utilizes advanced light source technology to enhance brightness, texture, and color contrast, potentially improving overall lesion visibility. Red dichromatic imaging is designed to enhance the visualization of deep blood vessels and is particularly beneficial for hemostasis during therapeutic endoscopic procedures and postprocedural monitoring. By filtering specific red wavelengths, RDI provides improved contrast of blood vessels against surrounding tissues, which aids in the identification of bleeding sources, including active spurting or oozing points. Recent studies have demonstrated its utility in improving the efficiency and accuracy of endoscopic hemostasis, especially in colorectal and upper gastrointestinal bleeding^9^. In addition to improving the visibility of bleeding points, RDI displays blood in a yellowish hue rather than a bright red, which may help reduce visual stress during endoscopic procedures. Given that red is psychologically associated with alarm and tension, the softer coloration in RDI may promote a calmer operating environment and aid in maintaining the endoscopist's composure, especially during active bleeding.

### Differences between NBI and BLI

Blue laser imaging and LCI utilize specific laser wavelengths to enhance mucosal details. Blue laser imaging provides a high contrast between neoplastic and non‐neoplastic tissues, particularly in magnified views. Similar to NBI, BLI uses laser‐based narrow‐band illumination with different wavelength combinations (410 nm for BLI vs. 415 nm for NBI) to optimize mucosal and vascular contrast. Some studies have suggested that compared with NBI, BLI offers superior brightness and improved image clarity in deeper mucosal layers, potentially enhancing lesion characterization. However, further comparative studies are required to determine the clinical advantages.

## DIAGNOSTIC ACCURACY OF IEE

### Detection capabilities

Here, we summarize studies that evaluated the ability of IEE to improve lesion detection rates, particularly in comparison with WLI. Numerous studies have assessed the effectiveness of various IEE modalities in detecting colorectal lesions. Table 1 provides a summary of key studies that compared different IEE techniques and their diagnostic performances.

**TABLE 1:** Key studies on image‐enhanced endoscopy detection capabilities.

Study	Comparison of modalities	Main findings
Leung et al. (2014)	NBI versus HD‐WLI	NBI improves adenoma detection rate (ADR).
Atkinson et al. (2019)	NBI versus WLI (meta‐analysis)	NBI enhances neoplasia detection, particularly in the proximal colon.
Hasegawa et al. (2021)	LCI versus WLI (tandem study)	LCI significantly increases neoplasm detection.
Paggi et al. (2020)	LCI versus WLI	LCI improves lesion detection in a screening program.
Fujimoto et al. (2018)	LCI versus WLI	LCI enhances the detection of sessile serrated adenomas/polyps (SSA/Ps).
Staudenmann et al. (2022)	NBI versus WLI (multicenter RCT)	NBI improves SSL detection, but the advantage over WLI is marginal.

Abbreviations: BLI, blue laser imaging; LCI, linked color imaging; NBI, narrow‐band imaging; SSL, sessile serrated lesion; TXI, texture and color enhancement imaging; WLI, white‐light imaging.

Several randomized controlled trials have demonstrated that NBI improves adenoma detection rates compared with WLI. Atkinson et al. conducted an individual patient data meta‐analysis of randomized trials and confirmed that NBI enhanced neoplastic lesion detection, particularly in the proximal colon^19^. Similarly, Leung et al. found that high‐definition NBI increased the detection of colorectal adenomas compared with high‐definition WLI^20^.

In addition to NBI, LCI has emerged as a promising technique for improving lesion visibility. Hasegawa et al. demonstrated in a tandem colonoscopy study that LCI significantly enhanced the detection of colorectal neoplasms compared with standard WLI^21^. A multicenter randomized trial by Paggi et al. further confirmed the superiority of LCI over WLI in an organized CRC screening program^22^.

The detection of sessile serrated lesions (SSLs) remains challenging because of their flat morphology and subtle color differences from the surrounding mucosa. Studies have shown that IEE techniques can improve SSL detection. However, the optimal modality remains unclear. Fujimoto et al. demonstrated that LCI enhances the detection of sessile serrated adenomas/polyps^23^. Similarly, Staudenmann et al. compared NBI with WLI in a prospective randomized multicenter study, suggesting that although NBI improves SSL detection, its advantage over WLI is not as pronounced as that of conventional adenomas^24^.

Although these findings support the use of IEE for lesion detection, direct comparisons among different IEE modalities remain limited. Most studies have focused on IEE versus WLI, rather than evaluating the relative superiority of NBI, LCI, and TXI. Further randomized trials comparing these modalities are required to establish definitive treatment standards. Additionally, for difficult‐to‐detect SSLs, chromoendoscopy with indigo carmine remains a valuable adjunct to improve contrast and lesion visualization.

### Invasion depth assessment

Accurate assessment of invasion depth is critical for determining an appropriate treatment strategy for early CRC. IEE techniques, particularly NBI magnification, chromoendoscopy, and pit pattern analysis, have been extensively studied because of their ability to predict deep submucosal invasion. Additionally, EUS‐ and AI‐assisted diagnostics have been explored as complementary methods for evaluating invasion depth. Table 2 summarizes key studies that have evaluated these modalities.

**TABLE 2:** Key studies on image‐enhanced endoscopy‐based invasion depth assessment

Study	Modality	Main findings
Kudo et al. (1994, 1996)	Pit pattern analysis	First classification of colorectal lesions using magnifying chromoendoscopy
Matsuda et al. (2008)	Chromoendoscopy	Validated pit pattern analysis for depth assessment
Ikematsu et al. (2010)	NBI magnification	Capillary pattern type IIIA/IIIB correlated with superficial versus deep invasion
Sano et al. (2016)	JNET classification	Standardized NBI‐based classification for colorectal neoplasia
Kobayashi et al. (2019)	JNET validation	Large‐scale clinical study confirming JNET diagnostic accuracy
Shimura et al. (2014)	EUS versus chromoendoscopy	Comparable accuracy in T1a versus T1b cancer diagnosis
Fu et al. (2008)	EUS versus NBI	Similar performance, but EUS had greater operator variability
Misawa et al. (2016)	AI‐assisted diagnosis	First study demonstrating AI's potential in invasion depth assessment
Mori et al. (2018)	AI in real‐time	AI‐assisted diagnosis improved reproducibility and accuracy

Abbreviations: AI, artificial intelligence; CONNECT, COlorectal NEoplasia Endoscopic Classification to Choose the Treatment; EUS, endoscopic ultrasound; JNET, Japan NBI Expert Team classification; NBI, narrow‐band imaging.

### IEE‐based depth diagnosis

IEE plays a crucial role in improving endoscopic differentiation between intramucosal neoplasms and deeply invasive cancers. Kudo et al. introduced pit pattern analysis using magnifying endoscopy, providing a foundation for differentiating between non‐invasive and invasive colorectal lesions.^25^, ^26^. Subsequent studies, such as that by Matsuda et al., validated the effectiveness of chromoendoscopy with crystal violet staining, demonstrating its high accuracy in predicting submucosal invasion^27^.

The advent of NBI magnification has further refined the invasion depth assessment. Ikematsu et al. proposed a classification system based on capillary pattern types IIIA and IIIB, correlating with superficial and deep submucosal invasion, respectively^28^. This was followed by the development of the Japan NBI Expert Team (JNET) classification by Sano et al., which standardizes NBI‐based differentiation of superficial colorectal lesions^29^. A large‐scale clinical validation by Kobayashi et al. confirmed the diagnostic utility of JNET in real‐world practice, particularly in distinguishing type 2B (indicating possible deep invasion) from type 3 (overt invasive carcinoma)^30^.

Despite these advancements, NBI alone has limitations in accurately differentiating type 2B from type 3 lesions. In cases in which uncertainty remains, BLI magnification and LCI have been explored as alternative modalities. Yoshida et al. demonstrated that BLI can enhance the visualization of vascular structures, aiding in more precise invasion depth estimation.^31^, ^32^. However, direct comparisons between these IEE techniques remain limited, and standardized diagnostic criteria for non‐NBI‐based IEE are still under development.

### Comparison with EUS and AI‐assisted diagnosis

**FIGURE 3:**
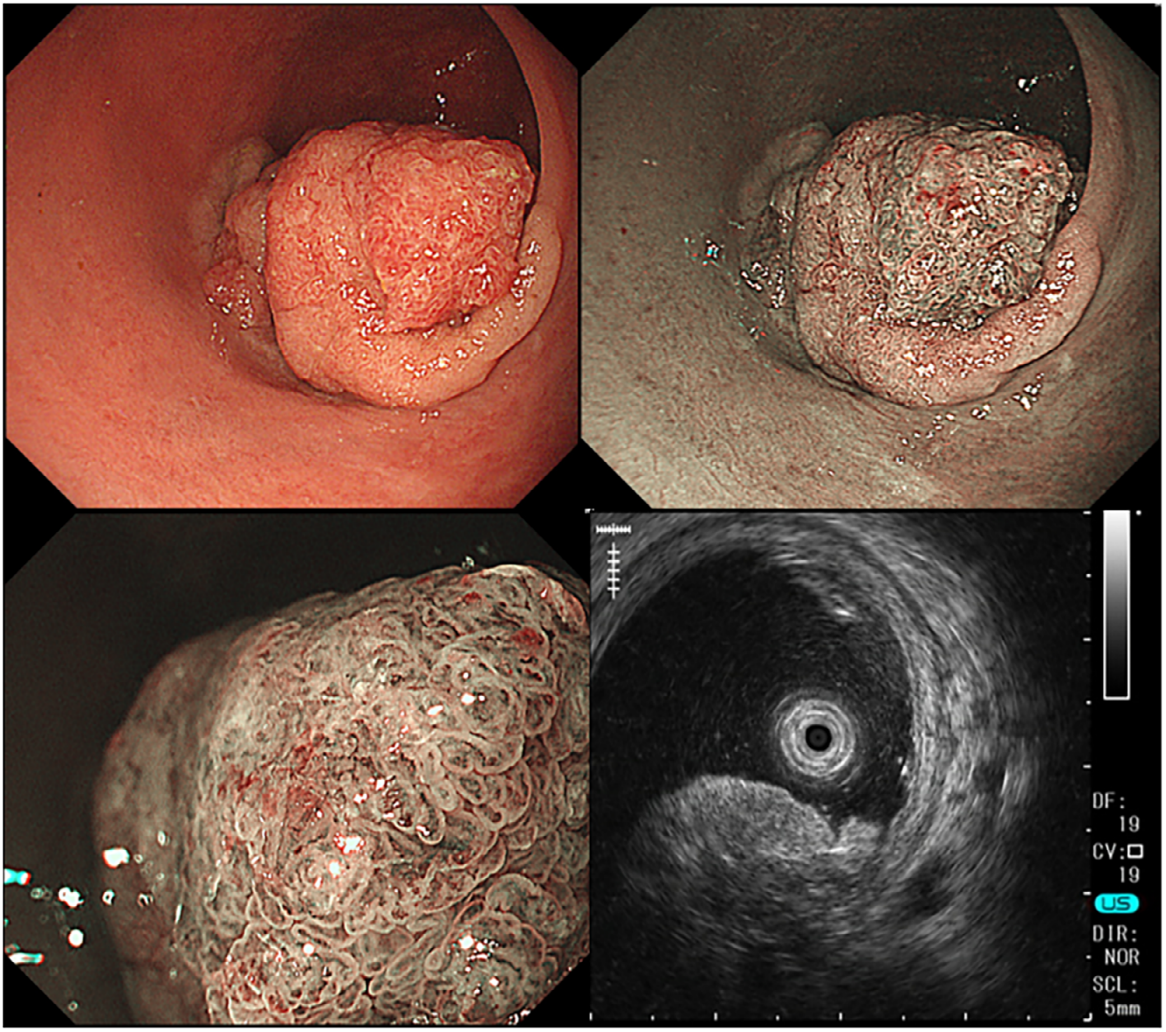
Endoscopic and endoscopic ultrasound assessment of an elevated colorectal cancer lesion. Although surface evaluation alone was insufficient to confirm deep invasion due to the absence of exposed highly atypical areas, endoscopic ultrasound assessment confirmed muscularis propria invasion, leading to a decision for surgical treatment. Pathological examination confirmed a pT2 depth of invasion.

Although IEE remains the primary method for assessing invasion depth, EUS has been explored as a complementary technique. Shimura et al. reported a prospective study comparing EUS and magnifying chromoendoscopy and found comparable accuracy in distinguishing between T1a (superficial submucosal invasion) and T1b (deep submucosal invasion) cancers^33^. Similarly, Fu et al. reported that EUS and NBI magnification yielded similar diagnostic performance, although EUS was more prone to operator variability^34^. Figure 3 shows an example of an elevated CRC lesion with suspected deep invasion. Although surface evaluation alone was insufficient to confirm deep invasion owing to the absence of exposed highly atypical areas, EUS assessment confirmed muscularis propria invasion, leading to the decision for surgical treatment. Pathological examination confirmed pT2 depth of invasion.

More recently, AI‐assisted diagnostics have shown promise in improving invasion depth estimation. Misawa et al. demonstrated that a deep learning model could differentiate between invasive and non‐invasive lesions with high accuracy,^35^. whereas Mori et al. reported the real‐time use of AI in endoscopic lesion characterization^36^. Although still in its early stages, AI has the potential to enhance objectivity and reproducibility in in‐depth diagnoses, addressing some of the limitations associated with subjective IEE interpretations.

In summary, IEE techniques, particularly NBI magnification and pit pattern analysis, remain the gold standards for invasion depth assessment. However, EUS serves as a useful adjunct in select cases and AI is an emerging tool that may further enhance diagnostic precision in the near future. Future studies should focus on standardizing the criteria across different IEE modalities and integrating AI‐assisted decision support into clinical practice.

In addition to classification systems that focus on histopathologic prediction, such as JNET, the CONNECT classification (COlorectal NEoplasia Endoscopic Classification to Choose the Treatment) has recently been introduced to facilitate direct treatment decision‐making^37^. This classification stratifies lesions into five categories based on macroscopic morphology and clinical risk assessment, guiding whether endoscopic mucosal resection, ESD, or surgical resection is appropriate. CONNECT is particularly valuable in ambiguous cases such as JNET type 2B lesions or large laterally spreading tumors (LSTs), where precise endoscopic prediction of pathology is challenging. It can be applied even without magnified observation and may thus complement existing IEE‐based diagnostic systems in real‐world clinical practice. Table 3 compares the JNET and CONNECT classifications in terms of their conceptual frameworks, clinical applications, and treatment implications.

**TABLE 3:** Key differences between the Japan NBI Expert Team and COlorectal NEoplasia Endoscopic Classification to Choose the Treatment classifications for colorectal lesions, highlighting their conceptual focus, required endoscopic techniques, and clinical applicability in colorectal lesion management.

Aspect	JNET classification	CONNECT classification
**Primary purpose**	Histological prediction (diagnosis‐oriented)	Direct treatment decision (treatment‐oriented)
**Basis of classification**	Magnified NBI assessment of vascular and surface patterns	Macroscopic morphology and clinical judgment
**Required equipment**	Magnifying endoscope with NBI	Standard endoscope (magnification not required)
**Typical application**	Precise diagnosis in expert settings	Practical use in general endoscopy or LSTs
**Relation to treatment**	Treatment selected based on predicted pathology	Treatment directly suggested by classification type

Abbreviations: CONNECT, COlorectal NEoplasia Endoscopic Classification to Choose the Treatment; JNET, Japan NBI Expert Team classification.

## ROLE OF EUS IN OVERCOMING THE LIMITATIONS OF IEE

IEE provides valuable information about mucosal patterns but lacks the capability to objectively assess the depth of tumor invasion. EUS has emerged as a complementary modality that allows real‐time evaluation of tumor depth and submucosal involvement. Combining EUS with IEE may enhance diagnostic accuracy, particularly in differentiating T1a from T1b lesions, which is critical for determining the need for additional lymph node evaluation or surgical intervention.

## ROLE OF PIT PATTERN DIAGNOSIS IN OVERCOMING THE LIMITATIONS OF IEE

In cases in which lesion characterization using NBI remains uncertain, pit pattern diagnosis using crystal violet staining plays a complementary role. Specifically, for lesions in which differentiation between JNET types 2B and 3 is challenging, the application of high‐magnification chromoendoscopy with crystal violet may be particularly useful. This approach enables a more precise assessment of the invasion depth and histological type, thereby guiding appropriate therapeutic decisions, such as en bloc resection or surgical intervention. An observational study demonstrated that pit pattern analysis with crystal violet significantly enhances the diagnostic accuracy for deeply invasive lesions, reducing both under‐ and over‐treatment^38^. Although crystal violet staining has come under scrutiny following health advisories (e.g., the 2019 report of Health Canada), no clinical evidence of carcinogenic risk has been reported for long‐term clinical use, supporting its continued role in selected difficult cases.

## IEE IN THE DIAGNOSIS OF UCAN

Ulcerative colitis‐associated neoplasia presents a significant clinical challenge owing to its multifocal nature, flat morphology, and frequent indistinct borders, making early detection difficult. Patients with long‐standing UC have an increased risk of developing colorectal neoplasia,^39^. which may progress to CRC if not identified and treated appropriately. Conventional WLI often fails to detect UCAN, leading to missed diagnoses and delayed intervention. Consequently, high‐definition chromoendoscopy or white‐light endoscopy with NBI is the preferred modality for the detection of UCAN^40^. The 2021 SCENIC update noted that high‐definition colonoscopy with targeted biopsies remains the primary recommended technique, as high‐definition chromoendoscopy requires a longer examination time^41^.

IEE, including NBI and TXI, have been investigated as a means of improving the detection of UCAN by enhancing the visibility of subtle mucosal changes. Studies have suggested that NBI can aid in identifying irregular or amorphous surface patterns and irregular or avascular vessel patterns, which indicate dysplasia in the setting of UC^42^. Additionally, TXI has been reported to enhance the color contrast and improve the delineation of UCAN's boundary^43^.

However, UCAN poses unique challenges because it often lacks well‐defined surface and vascular patterns, making it difficult to classify using standard IEE‐based systems such as JNET. Therefore, the sensitivity of JNET type 3 for UCAN has been reported to be only 25%, although its specificity was 100%^44^. Additionally, chronic inflammation can obscure neoplastic changes, reducing the effectiveness of IEE in distinguishing dysplastic from nondysplastic lesions in UC^45^. Notably, regenerative mucosa can exhibit type III_L_ and IV pit patterns, suggesting that such mucosal changes may reduce the specificity of IEE for UCAN detection^46^.

To address these limitations, recent studies have suggested that AI‐assisted IEE may further refine UCAN detection by providing real‐time analysis of suspicious areas^47^. Future studies should focus on integrating IEE, AI algorithms, and molecular biomarkers to develop comprehensive diagnostic strategies for UCAN. Standardizing surveillance protocols and optimizing imaging techniques are critical for improving early detection and reducing the risk of CRC in patients with UC.

## CONCLUSION

IEE has significantly improved the detection, characterization, and management of colorectal lesions by enhancing mucosal visualization and vascular pattern differentiation. However, its inherent limitations in assessing invasion depth necessitate the integration of complementary modalities, such as EUS, AI‐assisted diagnostics, and pit pattern analysis using crystal violet staining, to improve clinical decision‐making.

Recent advancements, including the incorporation of TXI‐ and AI‐assisted NBI, have demonstrated the potential for increasing diagnostic precision. As discussed earlier, while classifications such as JNET provide valuable histological prediction and aid in treatment planning, they may be limited in situations where surface features are ambiguous or magnification is unavailable. The CONNECT classification addresses this gap by directly linking endoscopic morphology to treatment recommendations, offering a complementary perspective that emphasizes practicality and immediate clinical decision‐making. The CONNECT classification also represents a new approach for refining lesion categorization; however, further validation is required in broader clinical settings. Despite these innovations, distinguishing JNET type 2A from 2B and type 2B from 3 remains challenging, highlighting the need for further studies to enhance diagnostic reliability and treatment selection.

The continued development of hybrid diagnostic strategies that integrate IEE, EUS, and AI‐driven analytics has the potential to optimize CRC screening and management. Standardizing classification criteria and refining imaging modalities are critical for improving diagnostic accuracy and ensuring optimal patient outcomes in endoscopic practice. For cases in which IEE classification alone is inconclusive, especially for JNET type 2B and type 3 lesions, crystal violet staining, and pit pattern diagnosis provide additional diagnostic accuracy. High‐magnification chromoendoscopy with crystal violet staining enables precise differentiation between neoplastic and non‐neoplastic lesions, allowing for more informed treatment selection.

## CONFLICT OF INTEREST STATEMENT

None.

## ETHICS STATEMENT

Not applicable.

## References

[1] Siegel RL , Giaquinto AN , Jemal A . Cancer statistics, 2024. CA Cancer J Clin 2024; 74: 12–49. 38230766 10.3322/caac.21820

[2] Rex DK , Boland CR , Dominitz JA *et al*. Colorectal Cancer Screening: Recommendations for Physicians and Patients From the U.S. Multi‐Society Task Force on Colorectal Cancer. Gastroenterology 2017; 153: 307–23. 28600072 10.1053/j.gastro.2017.05.013

[3] Jahn B , Bundo M , Arvandi M *et al*. One in three adenomas could be missed by white‐light colonoscopy – Findings from a systematic review and meta‐analysis. BMC Gastroenterol 2025; 25: 170. 40082770 10.1186/s12876-025-03679-4 PMC11908064

[4] East JE , Vleugels JL , Roelandt P *et al*. Advanced endoscopic imaging: European Society of Gastrointestinal Endoscopy (ESGE) technology review. Endoscopy 2016; 48: 1029–45. 27711949 10.1055/s-0042-118087

[5] McGill SK , Evangelou E , Ioannidis JP , Soetikno RM , Kaltenbach T . Narrow band imaging to differentiate neoplastic and non‐neoplastic colorectal polyps in real time: A meta‐analysis of diagnostic operating characteristics. Gut 2013; 62: 1704–13. 23300139 10.1136/gutjnl-2012-303965 PMC3841766

[6] Ikematsu H , Sakamoto T , Togashi K *et al*. Detectability of colorectal neoplastic lesions using a novel endoscopic system with blue laser imaging: A multicenter randomized controlled trial. Gastrointest Endosc 2017; 86: 386–94. 28147226 10.1016/j.gie.2017.01.017

[7] Suzuki T , Hara T , Kitagawa Y *et al*. Linked‐color imaging improves endoscopic visibility of colorectal nongranular flat lesions. Gastrointest Endosc 2017; 86: 692–7. 28193491 10.1016/j.gie.2017.01.044

[8] Yoshida N , Inoue K , Dohi O *et al*. Analysis of texture and color enhancement imaging for improving the visibility of non‐polypoid colorectal lesions. Dig Dis Sci 2022; 67: 5657–65. 35318554 10.1007/s10620-022-07460-5

[9] Uraoka T , Igarashi M . Development and clinical usefulness of a unique red dichromatic imaging technology in gastrointestinal endoscopy: A narrative review. Therap Adv Gastroenterol 2022; 15: 17562848221118302. 10.1177/17562848221118302 PMC9445450 36082177

[10] Saito Y , Sakamoto T , Fukunaga S , Nakajima T , Kiriyama S , Matsuda T . Endoscopic submucosal dissection (ESD) for colorectal tumors. Dig Endosc 2009; 21 (Suppl 1): S7–12. 19691740 10.1111/j.1443-1661.2009.00870.x

[11] Tanaka S , Terasaki M , Kanao H , Oka S , Chayama K Current status and future perspectives of endoscopic submucosal dissection for colorectal tumors. Dig Endosc 2012; 24: 73–9. 22533757 10.1111/j.1443-1661.2012.01252.x

[12] Ohata K , Kobayashi N , Sakai E *et al*. Long‐term outcomes after endoscopic submucosal dissection for large colorectal epithelial neoplasms: A prospective, multicenter, cohort trial from Japan. Gastroenterology 2022; 163: 1423–34.e2. 35810779 10.1053/j.gastro.2022.07.002

[13] Cârţână ET , Pârvu D , Săftoiu A . Endoscopic ultrasound: Current role and future perspectives in managing rectal cancer patients. J Gastrointestin Liver Dis 2011; 20: 407–13. 22187707

[14] Chen TH , Lin CJ , Wu RC . The application of miniprobe ultrasonography in the diagnosis of colorectal subepithelial lesions. Chang Gung Med J 2010; 33: 380–8. 20804667

[15] Minami S , Saso K , Miyoshi N *et al*. Diagnosis of depth of submucosal invasion in colorectal cancer with AI Using deep learning. Cancers 2022; 14: 5361. 36358780 10.3390/cancers14215361 PMC9656054

[16] Yao L , Lu Z , Yang G *et al*. Development and validation of an artificial intelligence‐based system for predicting colorectal cancer invasion depth using multi‐modal data. Dig Endosc 2023; 35: 625–35. 36478234 10.1111/den.14493

[17] Rutter MD , Saunders BP , Wilkinson KH *et al*. Thirty‐years analysis of a colonoscopic surveillance program for neoplasia in ulcerative colitis. Gastroenterology 2006; 130: 1030–8. 16618396 10.1053/j.gastro.2005.12.035

[18] Takabayashi K , Kato M , Kanai T . Clinical usefulness of image‐enhanced endoscopy for the diagnosis of ulcerative colitis‐associated neoplasia. DEN Open 2024; 4: e325. 38188357 10.1002/deo2.325 PMC10771229

[19] Atkinson NSS , Ket S , Bassett P *et al*. Narrow‐band imaging for detection of neoplasia at colonoscopy: A meta‐analysis of data from individual patients in randomized controlled trials. Gastroenterology 2019; 157: 462–71. 30998991 10.1053/j.gastro.2019.04.014

[20] Leung WK , Lo OS , Liu KS *et al*. Detection of colorectal adenoma by narrow band imaging (HQ190) vs. high‐definition white light colonoscopy: A randomized controlled trial. Am J Gastroenterol 2014; 109: 855–63. 24751581 10.1038/ajg.2014.83

[21] Hasegawa I , Yamamura T , Suzuki H *et al*. Detection of Colorectal neoplasms using linked color imaging: A prospective, randomized, tandem colonoscopy trial. Clin Gastroenterol Hepatol 2021; 19: 1708–16.e4. 33839277 10.1016/j.cgh.2021.04.004

[22] Paggi S , Radaelli F , Senore C *et al*. Linked‐color imaging versus white‐light colonoscopy in an organized colorectal cancer screening program. Gastrointest Endosc 2020; 92: 723–30. 32502550 10.1016/j.gie.2020.05.044

[23] Fujimoto D , Muguruma N , Okamoto K *et al*. Linked color imaging enhances endoscopic detection of sessile serrated adenoma/polyps. Endosc Int Open 2018; 6: E322–34. 29527554 10.1055/s-0043-124469 PMC5842067

[24] Staudenmann D , Liu K , Varma P *et al*. Narrow band imaging versus white light for detecting sessile serrated lesion: A prospective randomized multicenter study. DEN Open 2021; 2: e44. 35310703 10.1002/deo2.44 PMC8828189

[25] Kudo S . Endoscopic mucosal resection of flat and depressed types of early colorectal cancer. Endoscopy 1993; 25: 455–61. 8261988 10.1055/s-2007-1010367

[26] Kudo S , Tamura S , Nakajima T , Yamano H , Kusaka H , Watanabe H Diagnosis of colorectal tumorous lesions by magnifying endoscopy. Gastrointest Endosc 1996; 44: 8–14. 8836710 10.1016/s0016-5107(96)70222-5

[27] Matsuda T , Fujii T , Saito Y *et al*. Efficacy of the invasive/non‐invasive pattern by magnifying chromoendoscopy to estimate the depth of invasion of early colorectal neoplasms. Am J Gastroenterol 2008; 103: 2700–6. 18853968 10.1111/j.1572-0241.2008.02190.x

[28] Ikematsu H , Matsuda T , Emura F *et al*. Efficacy of capillary pattern type IIIA/IIIB by magnifying narrow band imaging for estimating depth of invasion of early colorectal neoplasms. BMC Gastroenterol 2010; 10: 33. 20346170 10.1186/1471-230X-10-33 PMC2868042

[29] Sano Y , Tanaka S , Kudo SE *et al*. Narrow‐band imaging (NBI) magnifying endoscopic classification of colorectal tumors proposed by the Japan NBI Expert Team. Dig Endosc 2016; 28: 526–33. 26927367 10.1111/den.12644

[30] Kobayashi S , Yamada M , Takamaru H *et al*. Diagnostic yield of the Japan NBI Expert Team (JNET) classification for endoscopic diagnosis of superficial colorectal neoplasms in a large‐scale clinical practice database. United European Gastroenterol J 2019; 7: 914–23. 10.1177/2050640619845987 PMC6683640 31428416

[31] Yoshida N , Hisabe T , Inada Y *et al*. The ability of a novel blue laser imaging system for the diagnosis of invasion depth of colorectal neoplasms. J Gastroenterol 2014; 49: 73–80. 23494646 10.1007/s00535-013-0772-7

[32] Yoshida N , Yagi N , Inada Y *et al*. Ability of a novel blue laser imaging system for the diagnosis of colorectal polyps. Dig Endosc 2014; 26: 250–8. 23731034 10.1111/den.12127

[33] Shimura T , Ebi M , Yamada T *et al*. Magnifying chromoendoscopy and endoscopic ultrasonography measure invasion depth of early‐stage colorectal cancer with equal accuracy on the basis of a prospective trial. Clin Gastroenterol Hepatol 2014; 12: 662–8.e1–2. 10.1016/j.cgh.2013.06.022 23872238

[34] Fu KI , Kato S , Sano Y *et al*. Staging of early colorectal cancers: Magnifying colonoscopy versus endoscopic ultrasonography for estimation of depth of invasion. Dig Dis Sci 2008; 53: 1886–92. 18080834 10.1007/s10620-007-0104-y

[35] Misawa M , Kudo SE , Mori Y *et al*. Accuracy of computer‐aided diagnosis based on narrow‐band imaging endocytoscopy for diagnosing colorectal lesions: Comparison with experts. Int J Comput Assist Radiol Surg 2017; 12: 757–66. 28247214 10.1007/s11548-017-1542-4

[36] Mori Y , Kudo SE , Misawa M *et al*. Real‐time use of artificial intelligence in identification of diminutive polyps during colonoscopy: A prospective study. Ann Intern Med 2018; 169: 357–66. 30105375 10.7326/M18-0249

[37] Brule C , Pioche M , Albouys J *et al*. The COlorectal NEoplasia Endoscopic Classification to Choose the Treatment classification for identification of large laterally spreading lesions lacking submucosal carcinomas: A prospective study of 663 lesions. United European Gastroenterol J 2022; 10: 80–92. 10.1002/ueg2.12194 PMC8830277 35089651

[38] Sakamoto T , Nakajima T , Matsuda T *et al*. Comparison of the diagnostic performance between magnifying chromoendoscopy and magnifying narrow‐band imaging for superficial colorectal neoplasms: An online survey. Gastrointest Endosc 2018; 87: 1318–23. 29309778 10.1016/j.gie.2017.12.021

[39] Shah SC , Itzkowitz SH . Colorectal cancer in inflammatory bowel disease: Mechanisms and management. Gastroenterology 2022; 162: 715–30.e3. 34757143 10.1053/j.gastro.2021.10.035 PMC9003896

[40] Rubin DT , Ananthakrishnan AN , Siegel CA , Sauer BG , Long MD . ACG clinical guideline: Ulcerative colitis in adults. Am J Gastroenterol 2019; 114: 384–413. 30840605 10.14309/ajg.0000000000000152

[41] Kiesslich R SCENIC update 2021: Is chromoendoscopy still standard of care for inflammatory bowel disease surveillance? Gastrointest Endosc 2022; 95: 38–41. 34801222 10.1016/j.gie.2021.10.009

[42] Nishiyama S , Oka S , Tanaka S *et al*. Clinical usefulness of narrow band imaging magnifying colonoscopy for assessing ulcerative colitis‐associated cancer/dysplasia. Endosc Int Open 2016; 4: E1183–7. 27853744 10.1055/s-0042-116488 PMC5110355

[43] Takabayashi K , Kato M , Sugimoto S , Yahagi N , Kanai T . Texture and color enhancement imaging in combination with indigo carmine dye spraying to highlight the border of flat ulcerative colitis‐associated neoplasia. Gastrointest Endosc 2022; 95: 1273–5. 35181337 10.1016/j.gie.2022.02.011

[44] Kawasaki K , Nakamura S , Esaki M *et al*. Clinical usefulness of magnifying colonoscopy for the diagnosis of ulcerative colitis‐associated neoplasia. Dig Endosc 2019; 31: 36–42. 30994234 10.1111/den.13382

[45] Akiyama S , Sakamoto T , Steinberg JM , Saito Y , Tsuchiya K . Evolving roles of magnifying endoscopy and endoscopic resection for neoplasia in inflammatory bowel diseases. World J Gastrointest Oncol 2022; 14: 646–53. 35321277 10.4251/wjgo.v14.i3.646 PMC8919023

[46] Hata K , Watanabe T , Motoi T , Nagawa H . Pitfalls of pit pattern diagnosis in ulcerative colitis‐associated dysplasia. Gastroenterology 2004; 126: 374–6. 14753219 10.1053/j.gastro.2003.05.020

[47] Kudo SE , Maeda Y , Ogata N *et al*. Combined endocytoscopy with pit pattern diagnosis in ulcerative colitis‐associated neoplasia: Pilot study. Dig Endosc 2022; 34: 133–43. 33641190 10.1111/den.13964

